# Dynamical Characterization of Plates Containing Plane Cracks with Functional Gradient Materials

**DOI:** 10.3390/ma18163868

**Published:** 2025-08-18

**Authors:** Gen Liu, An Xi, Yunchao Qi, Wenju Han

**Affiliations:** 1 School of Advanced Manufacturing Technology, Guangdong Mechanical and Electrical Polytechnic, Guangzhou 510550, China; liugen1991@hotmail.com; 2 China CEPREI Laboratory, Institute of Software and Systems, Guangzhou 511370, China; xian@ceprei.com; 3 Ministry of Industry and Information Technology Key Laboratory of Industrial Software Engineering Application Technology, Guangzhou 511370, China; 4 School of Aerospace Engineering, North University of China, Taiyuan 030051, China

**Keywords:** functionally graded material plate, vibration characteristics, Ritz method, planar crack, asymmetrical phenomena

## Abstract

This study develops a vibration model for functionally graded material (FGM) plates with embedded planar cracks. Based on thin plate theory and von Kármán-type geometric nonlinear strain assumptions, the kinetic and potential energies of each region are derived. Displacement field trial functions are constructed according to boundary conditions, and the Ritz method is employed to determine natural frequencies and vibration modes under small deformation conditions. The investigation focuses on how crack parameters and material gradient coefficients affect vibration characteristics in exponentially graded FGM plates. The results show that natural frequencies decrease with increasing crack length, while crack presence alters nodal line patterns and mode symmetry. During free vibration, the upper and lower surfaces of the crack region exhibit relative displacement. Material gradient effects induce thickness–direction asymmetry, causing non-uniform displacements between the plate’s upper and lower sections.

## 1. Introduction

Composite plate structures are widely used in the engineering field. However, damages such as cracks and delamination can significantly affect their mechanical properties. Scholars have conducted research on vibration, buckling, crack propagation, and other aspects of functionally graded material (FGM) plates and laminated composite plates, providing theoretical support for structural design, performance evaluation, and optimization.

Functionally graded materials are regarded as one of the most promising candidates for future advanced composites in many engineering sectors. However, FGMs are not immune to the occurrence of plane cracks (delamination), which can significantly reduce the stiffness and strength of the structures and affect their vibration characteristics. In terms of vibration characteristic analysis, focusing on FGM plates with cracks, Minh and Duc applied Shi’s third-order shear deformation theory and phase field theory to study the effect of cracks and thickness variation on the free vibration parameters of FGM plates under temperature [1], revealing the influence laws of crack parameters (length, angle, etc.), temperature, and material properties on the vibration frequency. Burlayenko et al. conducted a three-dimensional modeling study on the free vibrations of functionally graded material (FGM) sandwich plates subjected to thermal loads. They carried out a parametric study on frequency analysis by varying the volume fraction profile of the material and the temperature distribution across the thickness of the plate and explored in depth the effects of these factors on the vibration characteristics [2]. Obalareddy et al. employed the dynamic stiffness matrix (DSM) method to conduct a free vibration analysis of a functionally graded (FG) rotor-bearing system subjected to temperature gradients and explored its potential applications in FG rotors [3].

Gupta et al. proposed a non-classical analytical model for plates containing star-shaped cracks based on classical plate theory and modified couple stress theory [4], clarifying the influence of crack parameters on the fundamental frequency and the phenomenon of bending hardening/softening. Safaeifar adopted the variational iteration method (VIM), combined with the Hamilton principle and the Ritz numerical method, to analyze the vibration characteristics of FGM plates under axial and lateral loads [5], obtaining natural frequencies under different boundary conditions. For FGM plates with delamination effects, Wang et al. used the Chebyshev–Ritz method, considering the effects of exponential law distribution of materials, plane cracks, and delamination, and analyzed the kinetic and potential energies based on the region approach [6], explaining the influence of material distribution and delamination length ratio on vibration behavior. Mishra et al. adopted the finite element method and introduced functional materials to study their influence on the delamination effect and the dynamic response of the plate [7], proposing methods to control and reduce the delamination effect and optimizing the overall performance of FGM plates with delamination. For laminated composite plates with damage, Li et al. applied the extended layer-wise method (XLWM), simulated the damage through displacement assumptions and XFEM discretization, and analyzed the static, free vibration, and transient responses of laminated composite plates with damage [8], accurately predicting the displacement and stress fields of damaged FGM plates and verifying the effectiveness of the method. Sahoo et al. used the higher-order shear deformation theory and the finite element method for modeling, combined with the variational method and the Hamilton principle to obtain the governing equations [9], analyzing the influence of delamination on the vibration behavior of laminated composite plates. Huang et al. assumed the delamination position, modeled with the improved layer-wise theory and the finite element method, and considered the foundation parameters and boundary conditions to study the nonlinear vibration behavior [10], investigating the influence of delamination on the nonlinear fundamental frequency and providing a basis for damage detection.

In the aspect of buckling characteristic analysis with regard to cracked FGM plates, Zenkour and Doan studied the buckling of cracked FGM plates based on the higher-order shear deformation theory, combined with the phase field theory and the finite element method, considering the delamination of the elastic foundation [11], clarifying the influence of delamination area and crack length on the buckling load and mode. Taheri and Memarzadeh calculated the buckling of CNTRC plates with off-center cracks under shear loading using the extended finite element method (XFEM) [12], revealing the influence laws of CNT parameters and crack parameters on the shear buckling capacity. Zhong analyzed the buckling load based on the generalized third-order plate theory and non-local theory, using the surface elasticity theory and the Navier solution method, considering the surface effect [13], obtaining the relationship between the surface effect and the plate parameters, and discussing its influence on the buckling characteristics. For FGM plates with delamination, Karataş studied the buckling delamination of rectangular orthotropic thick plates with embedded cracks under axial compressive force within the framework of the three-dimensional geometrically nonlinear elasticity theory using the three-dimensional finite element method [14], evaluating the influence of material and geometric parameters on the critical buckling force. Ertenli and Esen studied the influence of porous layers on the thermo-mechanical buckling of sandwich porous plates using a new sinusoidal higher-order shear theory [15], clarifying the influence of porosity, temperature increase, and material gradient index on the thermal buckling load. For laminated composite plates with damage, Kim et al. introduced local and zero stiffness models, determined the overall stiffness based on the first-order shear deformation theory (FSDT) and the classic laminate theory (CLT), and analyzed the bending and buckling problems [16], providing a method for determining the overall stiffness of damaged plates. Juhász and Szekrényes used the Mindlin plate finite element method to simulate and verify through experiments the influence of delamination on the critical buckling force [17], confirming that the simulation model can predict the influence of delamination on the buckling force and discussing the generalization of the results. Kiani Y studied the thermal post buckling phenomenon based on the first-order shear deformation plate theory, considering the temperature-related properties and using the cylindrical arc-length technique [18], revealing that the structure is sensitive to the initial deflection caused by the thermal post buckling load and analyzing the influence of related factors.

In research on crack propagation and damage prediction for FGM plates, Li proposed the VCCT-XLWM method to predict delamination and transverse crack propagation based on XLWM and the virtual crack closure technique (VCCT) [19], effectively predicting the delamination and crack growth of laminated plates and shells. Li et al. extended the XLWM used for beams to plates, simulated delamination and cracks with specific functions, and calculated relevant parameters [20], accurately predicting the fields near the crack tip and delamination front and the crack growth angle. Nabil et al. compared different crack growth criteria and studied the crack propagation of FGM plates using advanced finite element technology and improved displacement extrapolation techniques [21], clarifying the accuracy and effectiveness of different criteria for predicting crack propagation paths. For laminated composite plates, Pokrovskii et al. proposed a method for evaluating the crack resistance of compressed composite plates with initial delamination based on the J-integral fracture criterion [22], calculated the fracture force, and compared it with the experimental data, with an error less than 10%, verifying the reliability of the method. McElroy et al. proposed a new test method that used ultrasonic and X-ray detection to record the damage process of CFRP plates and used it for model validation [23], providing an effective test means for studying the interaction between delamination and matrix cracks.

In other related research, for FGM plates, Zheng et al. proposed the hybrid meshless displacement discontinuity method (MDDM), using specific fundamental solutions and meshless methods to solve the problem of cracked FGM Reisner plates [24], verifying the accuracy of the MDDM method. Tran et al. used the moving element method (MEM), combined with the Mindlin plate theory and the virtual work principle, and considered the temperature influence to analyze the dynamics of FGM plates under moving loads [25], finding that the temperature has a significant influence on the dynamic response of FGM plates and the displacement increases with the increase of temperature. For laminated composite plates, Kim et al. studied the method for determining the local stiffness of cross-ply composite plates with delamination and matrix cracks, divided the region to calculate the strain energy [26], determined the local stiffness, and evaluated the effectiveness of the method based on crack modeling and local stiffness. Lu et al. developed the extended layer wise/solid-element (XLW/SE) method for stiffened composite plates with transverse cracks and delamination [27]. The XLW/SE method can accurately simulate damaged stiffened composite plates, which is in good agreement with the results of existing models. Xu et al. applied XLWM to the static analysis of laminated piezoelectric and composite plates with delamination, cracks, or debonding of piezoelectric patches [28], establishing a coupling analysis model for damaged plates with piezoelectric patches and considering various factors. Wu et al. developed several numerical analysis methods for damaged composite plates, such as the analysis methods for stiffened plates with piezoelectric patches and sandwich plates with piezoelectric sensors [29,30]. The developed numerical models can effectively analyze damaged composite plates and verify the accuracy of the methods. Ghazali et al. used regression and neural network methods, constructed a data set to evaluate the effectiveness of algorithms for detecting and locating delamination and cracks [31], and evaluated the effectiveness of machine learning algorithms for detecting and locating delamination and cracks. Wang et al. established a finite element model to study the influence of interfacial delamination on cracks in thick diamond-like carbon coatings under indentation [32], clarifying the influence of coating thickness and elastic modulus on interfacial delamination and cracks. Aylikci et al. studied the buckling delamination problem of piezoelectric sandwich plates with interface inner band cracks using the three-dimensional finite element method [33], determining the critical force of piezoelectric sandwich plates with interface inner band cracks, and analyzing the influence of parameters. Cui et al. established a model of a cantilevered plate with cracks and used specific functions and methods to study the stability of the plate under low-speed axial flow loading [34], revealing the influence law of cracks on the stability of the plate under low-speed axial flow loading. Khoram-Nejad et al. analyzed the influence of crack characteristics on the vibration behavior of post-buckled functionally graded plates and solved it with specific models and methods [35], providing a reference for the influence of crack characteristics on the vibration behavior of post-buckled functionally graded plates. In related research on thermal barrier coatings, Dong et al. studied the coupling effect of delamination cracks and vertical cracks on the local phase transition of the ceramic top coating of thermal barrier coatings through gradient thermal cycling tests, using Raman spectroscopy and two-dimensional models [36], finding that the crack coupling effect increases the coating temperature, leading to serious sintering and phase transition, and clarifying its time-space characteristics. In related research on composite strips, Salunkhe et al. developed a method combining the cross-sectional model and one-dimensional theory, considered intramural and interlinear cracks, and used specific methods to simulate and study the influence of defects on the extension–twist coupling of thin retwisted composite strips [37], revealing the opposite influence of different types of defects on the trapeze effect, explaining the relationship with the cross-sectional coupling stiffness terms, and predicting the strip stiffness degradation. Xue [38,39,40] adopted a semi-analytical method (the Ritz method combined with locally admissible functions) to study the vibration localization of plate-type resonators, emphasizing the crucial role of non-orthogonality in modal coupling.

In this paper, the semi-analytical method is used to model the vibration of a plate containing a plane-cracked functional gradient material (FGM), where the plane crack is perpendicular to the thickness direction of the plate and penetrates through the plate width. The literature review results show that although there are already a large number of studies on the crack and fracture behavior analysis of functionally graded beams (FGM beams), there are fewer reports on the vibration characteristics of functionally graded structures with delamination defects. This study explores the influence of delamination defects (specifically, including their length and position parameters) on the natural frequency of exponentially graded beams. The analytical analysis results obtained in this study can provide a reliable benchmark verification basis for the finite element method (FEM) and other numerical solution methods.

## 2. Modeling

**Figure 1:**
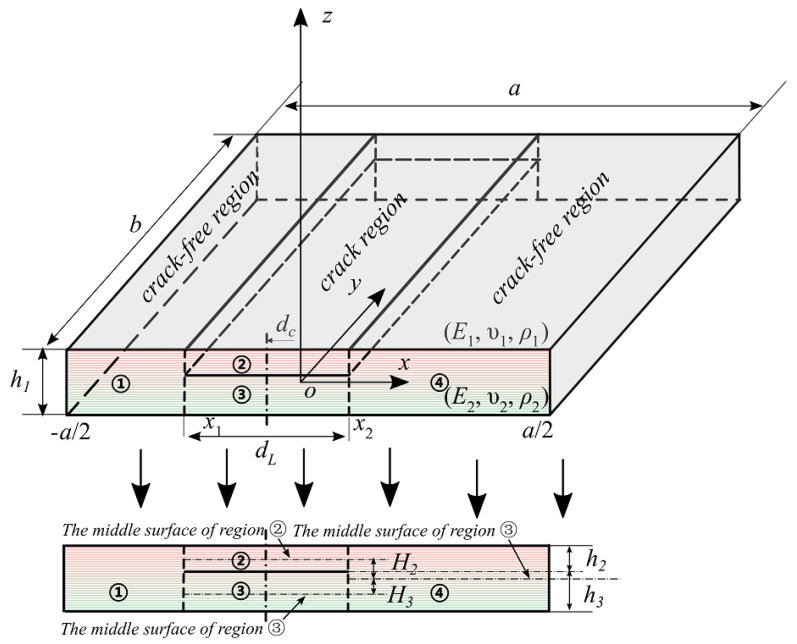
Geometry and dimension of an exponential functionally graded plate with a single planar crack.

As shown in Figure 1, an exponential functional material gradient plate containing a planar crack is considered. The length and width of the plate are a and b, respectively; the planar crack is parallel to the xy plane and runs across the width of the plate in the y-direction; the length of the crack is $d_{L}$; and the distance between the mid-point of the planar crack and the mid-point of the plate is $d_{c}$, and is referred to as the location of the crack. Due to the presence of the crack, the plate is divided into three regions in the x-direction: a region where the plane crack exists and two intact regions ① and ④, which are not related to the crack. The former is subdivided into two sub-regions ② and ③ in the plate thickness direction, which are connected to the intact regions ① and ④. The modulus of elasticity E, and the density ρ of the FGM plate are exponential functions of the thickness:

(1) $$E\left(z\right)=E_{0}e^{\beta z},\;\rho \left(z\right)=\rho_{0}e^{\beta z}$$

where β is the gradient parameter of the material, and $E_{0}$ and $\rho_{0}$ are the modulus of elasticity and density at the mid-face of the FGM plate, respectively. The modulus of elasticity, Poisson’s ratio, and density at the upper and lower surfaces of the plate can be expressed by $E_{1}$, $\upsilon_{1}$, $\rho_{1}$, $E_{2}$, $\upsilon_{2}$, and $\rho_{2}$. Since Poisson’s ratio varies less, it can be simplified to a constant υ. Figure 1 depicts the thicknesses of different sub-zones and their respective neutral surfaces.

## 3. Displacement Field of the Cracked Plate

For free vibration, the displacements at an arbitrary point on the sub-regions can be set as

(2) $$\left.\begin{array}{c} u_{0}^{\left(k\right)}\left(x,y,t\right)=u_{0}^{\left(k\right)}(x,y)e^{i\omega t}\\ v_{0}^{\left(k\right)}\left(x,y,t\right)=v_{0}^{\left(k\right)}\left(x,y\right)e^{i\omega t}\\ w_{0}^{\left(k\right)}\left(x,y,t\right)=w^{\left(k\right)}\left(x,y\right)e^{i\omega t} \end{array}\right\}$$

where k = 1, 2, 3, 4 represents the four sub-regions of the plate; $u_{0}^{\left(k\right)}$ and $v_{0}^{\left(k\right)}$ are the in-plane displacements on the mid-plane; and $w_{0}^{\left(k\right)}$ is the mid-plane transverse deflection.

Constructing the modal function of the displacement field based on the boundary conditions of the plate and the deformation coordination conditions in the cracked region is one of the key steps in this study.

The deformation coordination conditions for the in-plane displacement of a plate with a functional gradient material containing a plane crack are

(3) $$\left.\begin{array}{c} {\left.u^{\left(1\right)}\right|}_{x=x_{1}}={\left.u^{\left(2\right)}\right|}_{x=x_{1}}-{\left.H_{2}w_{,x}^{\left(1\right)}\right|}_{x=x_{1}}={\left.u^{\left(3\right)}\right|}_{x=x_{1}}+{\left.H_{3}w_{,x}^{\left(1\right)}\right|}_{x=x_{1}}\\ {\left.u^{\left(4\right)}\right|}_{x=x_{2}}={\left.u^{\left(2\right)}\right|}_{x=x_{2}}-{\left.H_{2}w_{,x}^{\left(4\right)}\right|}_{x=x_{2}}={\left.u^{\left(3\right)}\right|}_{x=x_{2}}+{\left.H_{3}w_{,x}^{\left(2\right)}\right|}_{x=x_{2}}\\ {\left.v^{\left(1\right)}\right|}_{x=x_{1}}={\left.v^{\left(2\right)}\right|}_{x=x_{1}}-{\left.H_{2}w_{,y}^{\left(1\right)}\right|}_{x=x_{1}}={\left.v^{\left(3\right)}\right|}_{x=x_{1}}+{\left.H_{3}w_{,y}^{\left(1\right)}\right|}_{x=x_{1}}\\ {\left.v^{\left(4\right)}\right|}_{x=x_{2}}={\left.v^{\left(2\right)}\right|}_{x=x_{2}}-{\left.H_{2}w_{,y}^{\left(4\right)}\right|}_{x=x_{2}}={\left.v^{\left(3\right)}\right|}_{x=x_{2}}+{\left.H_{3}w_{,y}^{\left(2\right)}\right|}_{x=x_{2}} \end{array}\right\}$$

The deformation coordination condition for out-of-plane displacements is

(4) $$\left.\begin{array}{c} {\left.w^{\left(1\right)}\right|}_{x=x_{1}}={\left.w^{\left(2\right)}\right|}_{x=x_{1}}={\left.w^{\left(3\right)}\right|}_{x=x_{1}}\\ {\left.w^{\left(4\right)}\right|}_{x=x_{2}}={\left.w^{\left(2\right)}\right|}_{x=x_{2}}={\left.w^{\left(3\right)}\right|}_{x=x_{2}}\\ {\left.w_{,x}^{\left(1\right)}\right|}_{x=x_{1}}={\left.w_{,x}^{\left(2\right)}\right|}_{x=x_{1}}={\left.w_{,x}^{\left(3\right)}\right|}_{x=x_{1}}\\ {\left.w_{,x}^{\left(4\right)}\right|}_{x=x_{2}}={\left.w_{,x}^{\left(2\right)}\right|}_{x=x_{2}}={\left.w_{,x}^{\left(3\right)}\right|}_{x=x_{2}} \end{array}\right\}$$

The method in this paper is applicable to a wide range of boundary conditions such as simply supported, cantilevered, and free. For the sake of simplicity, the two most common combinations of boundary conditions are chosen: one with one side fixed and other sides free (CFFF) and the other with opposite sides fixed and opposite sides free (CCFF).

For a plate solidly supported at *x* = $-\frac{a}{2}$ with other sides free (CFFF), the displacement boundary conditions are

(5) $$\left.\begin{array}{c} {\left.w^{\left(1\right)}\right|}_{x=-\frac{a}{2}}=0\\ {\left.w_{,x}^{\left(1\right)}\right|}_{x=-\frac{a}{2}}=0 \end{array}\right\}$$

(6) $$\left.\begin{array}{c} {\left.u^{\left(1\right)}\right|}_{x=-\frac{a}{2}}=0\\ {\left.v^{\left(1\right)}\right|}_{x=-\frac{a}{2}}=0 \end{array}\right\}$$

For the cases where x=−a/2 and x=a/2 are fixed and the other two sides are free (CCFF), the boundary conditions are

(7) $$\left.\begin{array}{c} {\left.w^{\left(1\right)}\right|}_{x=-\frac{a}{2}}={\left.w^{\left(4\right)}\right|}_{x=\frac{a}{2}}=0\\ {\left.w_{,x}^{\left(1\right)}\right|}_{x=-\frac{a}{2}}={\left.w_{,x}^{\left(4\right)}\right|}_{x=\frac{a}{2}}=0 \end{array}\right\}$$

(8) $$\left.\begin{array}{c} {\left.u^{\left(1\right)}\right|}_{x=-\frac{a}{2}}={\left.u^{\left(4\right)}\right|}_{x=\frac{a}{2}}=0\\ {\left.v^{\left(1\right)}\right|}_{x=-\frac{a}{2}}={\left.v^{\left(4\right)}\right|}_{x=\frac{a}{2}}=0 \end{array}\right\}$$

The Ritz method solution of the latter requires the selection of appropriate linearly independent and satisfying essential boundary conditions displacement field trial functions to ensure the accuracy of the solution. According to the deformation coordination conditions and boundary conditions of each sub-zone of the cracked plate, the appropriate trial function can be constructed. Considering the stability, efficiency, and convergence speed of the results, Chebyshev polynomials are selected as the test function to calculate the dynamic response of the cracked FGM plate [41].

Chebyshev polynomials are a kind of orthogonal polynomials, and the one-dimensional Chebyshev polynomial can be expressed as

(9) $$\Psi_{i}\left(\zeta \right)=L\left(\zeta \right)p_{i}\left(\zeta \right)\;(i=1,2,3\ldots )$$

Here, $L\left(\zeta \right)$ is the function associated with the boundary conditions such that each polynomial $\Psi_{i}\left(\zeta \right)$ satisfies arbitrary boundary conditions; $p_{i}\left(\zeta \right)$ is the basis function with the expression

(10) $$p_{i}\left(\zeta \right)=cos[(i-1)arccos(\zeta )]$$

The basis function in the x direction is obtained by making ζ in equation equal to 2x/a:

(11) $$X_{i}\left(x\right)=cos[(i-1)arccos(\frac{2x}{a})]$$

Similarly, the basis function in the y direction is obtained by making ζ=2y/b:

(12) $$Y_{j}\left(y\right)=\cos\left[\left(j-1\right)\arccos\left(\frac{2y}{b}\right)\right]$$

Under arbitrary boundary conditions, the in-plane displacement function for each sub-zone of a plate containing a gradient material with a crack function can be expressed as

(13) $$\left.\begin{array}{c} u^{\left(1\right)}=L_{u}^{\left(1\right)}\left(x,y\right)\sum_{i}^{I}\sum_{j}^{J}A_{ij}^{\left(1\right)}X_{i}\left(x\right)Y_{j}\left(y\right)\;\;\\ u^{\left(2\right)}=L_{u}^{\left(2\right)}\left(x,y\right)\sum_{i}^{I}\sum_{j}^{J}A_{ij}^{\left(2\right)}X_{i}\left(x\right)Y_{j}\left(y\right)+u^{\left(1\right)}+H_{2}^{u}\left(x,y\right)\\ u^{\left(3\right)}=L_{u}^{\left(3\right)}\left(x,y\right)\sum_{i}^{I}\sum_{j}^{J}A_{ij}^{\left(3\right)}X_{i}\left(x\right)Y_{j}\left(y\right)+u^{\left(1\right)}+H_{3}^{u}\left(x,y\right)\\ u^{\left(4\right)}=L_{u}^{\left(4\right)}\left(x,y\right)\sum_{i}^{I}\sum_{j}^{J}A_{ij}^{\left(1\right)}X_{i}\left(x\right)Y_{j}\left(y\right)\;\; \end{array}\right\}$$

(14) $$\left.\begin{array}{c} v^{\left(1\right)}=L_{v}^{\left(1\right)}\left(x,y\right)\sum_{i}^{I}\sum_{j}^{J}B_{ij}^{\left(1\right)}X_{i}\left(x\right)Y_{j}\left(y\right)\;\;\\ v^{\left(2\right)}=L_{v}^{\left(2\right)}\left(x,y\right)\sum_{i}^{I}\sum_{j}^{J}B_{ij}^{\left(2\right)}X_{i}\left(x\right)Y_{j}\left(y\right)+v^{\left(1\right)}+H_{2}^{v}\left(x,y\right)\\ v^{\left(3\right)}=L_{v}^{\left(3\right)}\left(x,y\right)\sum_{i}^{I}\sum_{j}^{J}B_{ij}^{\left(3\right)}X_{i}\left(x\right)Y_{j}\left(y\right)+v^{\left(1\right)}+H_{3}^{v}\left(x,y\right)\\ v^{\left(4\right)}=L_{v}^{\left(4\right)}\left(x,y\right)\sum_{i}^{I}\sum_{j}^{J}B_{ij}^{\left(1\right)}X_{i}\left(x\right)Y_{j}\left(y\right)\;\; \end{array}\right\}$$

Here, I and J are the number of terms taken by the basis functions in the x and y directions, respectively; $L_{u}^{\left(k\right)}$ and $L_{v}^{\left(k\right)}$ (*k* = 1, 2, 3, 4) are the functions related to the boundary conditions; ${\;H}_{2}^{u}\left(x\right),\;H_{3}^{u}\left(x\right),\;H_{2}^{v}\left(x\right)$ and $H_{3}^{v}\left(x\right)$ are obtained by the conditions of coordination of the deformation in the cracked region and the crack-free region, and they are

(15) $$\left.\begin{array}{c} H_{2}^{u}\left(x,y\right)=-H_{2}\left[\left(x_{1}-x\right){\left.w_{,x}^{\left(4\right)}\right|}_{x=x_{2}}+\left(x_{2}-x\right){\left.w_{,x}^{\left(1\right)}\right|}_{x=x_{1}}\right]/\left(x_{1}-x_{2}\right)\\ H_{3}^{u}\left(x,y\right)=H_{3}\left[\left(x_{1}-x\right){\left.w_{,x}^{\left(4\right)}\right|}_{x=x_{2}}+\left(x_{2}-x\right){\left.w_{,x}^{\left(1\right)}\right|}_{x=x_{1}}\right]/\left(x_{1}-x_{2}\right)\; \end{array}\right\}$$

(16) $$\left.\begin{array}{c} H_{2}^{v}\left(x,y\right)=-H_{2}\left[\left(x_{1}-x\right){\left.w_{,y}^{\left(4\right)}\right|}_{x=x_{2}}+\left(x_{2}-x\right){\left.w_{,y}^{\left(1\right)}\right|}_{x=x_{1}}\right]/\left(x_{1}-x_{2}\right)\\ H_{3}^{v}\left(x,y\right)=H_{3}\left[\left(x_{1}-x\right){\left.w_{,y}^{\left(4\right)}\right|}_{x=x_{2}}+\left(x_{2}-x\right){\left.w_{,y}^{\left(1\right)}\right|}_{x=x_{1}}\right]/\left(x_{1}-x_{2}\right)\; \end{array}\right\}$$

The out-of-plane displacement function for each sub-zone can be expressed as

(17) $$\left.\begin{array}{c} w^{\left(1\right)}=L_{w}^{\left(1\right)}\left(x,y\right)\sum_{i}^{I}\sum_{j}^{J}C_{ij}^{\left(1\right)}X_{i}\left(x\right)Y_{j}\left(y\right)\;\\ w^{\left(2\right)}=L_{w}^{\left(2\right)}\left(x,y\right)\sum_{i}^{I}\sum_{j}^{J}C_{ij}^{\left(2\right)}X_{i}\left(x\right)Y_{j}\left(y\right)+w^{\left(1\right)}\\ w^{\left(3\right)}=L_{w}^{\left(3\right)}\left(x,y\right)\sum_{i}^{I}\sum_{j}^{J}C_{ij}^{\left(3\right)}X_{i}\left(x\right)Y_{j}\left(y\right)+w^{\left(1\right)}\\ w^{\left(4\right)}=L_{w}^{\left(4\right)}\left(x,y\right)\sum_{i}^{I}\sum_{j}^{J}C_{ij}^{\left(1\right)}X_{i}\left(x\right)Y_{j}\left(y\right)\; \end{array}\right\}$$

Here, $L_{w}^{\left(k\right)}\;(k=1,2,3,4)$ is attached to the basis functions such that the out-of-plane displacement functions satisfy the corresponding boundary conditions.

For the boundary at x=−a/2 solidly supported and other three sides free (CFFF), 


(18) $$\left.\begin{array}{c} L_{u}^{\left(1\right)}\left(x,y\right)=L_{u}^{\left(4\right)}\left(x,y\right)=x+\frac{a}{2}\\ L_{u}^{\left(2\right)}\left(x,y\right)=(x-x_{1})(x-x_{2})\;\\ L_{u}^{\left(3\right)}\left(x,y\right)=(x-x_{1})(x-x_{2})\; \end{array}\right\}$$

(19) $$\left.\begin{array}{c} L_{v}^{\left(1\right)}\left(x,y\right)=L_{v}^{\left(4\right)}\left(x,y\right)=x+\frac{a}{2}\\ L_{v}^{\left(2\right)}\left(x,y\right)=(x-x_{1})(x-x_{2})\;\\ L_{v}^{\left(3\right)}\left(x,y\right)=(x-x_{1})(x-x_{2})\; \end{array}\right\}$$

(20) $$\left.\begin{array}{c} L_{w}^{\left(1\right)}\left(x,y\right)=L_{w}^{\left(4\right)}\left(x,y\right)={\left(x+\frac{a}{2}\right)}^{2}\\ L_{w}^{\left(2\right)}\left(x,y\right)={\left(x-x_{1}\right)}^{2}{\left(x-x_{2}\right)}^{2}\;\\ L_{w}^{\left(3\right)}\left(x,y\right)={\left(x-x_{1}\right)}^{2}{\left(x-x_{2}\right)}^{2}\; \end{array}\right\}$$

The boundary conditions at x=−a/2 and x=a/2 solidly supported and other two sides free (CCFF) can be similarly obtained as

(21) $$\left.\begin{array}{c} L_{u}^{\left(1\right)}\left(x,y\right)=L_{u}^{\left(4\right)}\left(x,y\right)=\left(x+\frac{a}{2}\right)\left(x-\frac{a}{2}\right)\\ L_{u}^{\left(2\right)}\left(x,y\right)=\left(x-x_{1}\right)\left(x-x_{2}\right)\;\\ L_{u}^{\left(3\right)}\left(x,y\right)=\left(x-x_{1}\right)\left(x-x_{2}\right)\; \end{array}\right\}$$

(22) $$\left.\begin{array}{c} L_{v}^{\left(1\right)}\left(x,y\right)=L_{v}^{\left(4\right)}\left(x,y\right)=\left(x+\frac{a}{2}\right)\left(x-\frac{a}{2}\right)\\ L_{v}^{\left(2\right)}\left(x,y\right)=\left(x-x_{1}\right)\left(x-x_{2}\right)\;\\ L_{v}^{\left(3\right)}\left(x,y\right)=\left(x-x_{1}\right)\left(x-x_{2}\right)\; \end{array}\right\}$$

(23) $$\left.\begin{array}{c} L_{w}^{\left(1\right)}\left(x,y\right)=L_{w}^{\left(4\right)}\left(x,y\right)={\left(x+\frac{a}{2}\right)}^{2}{\left(x-\frac{a}{2}\right)}^{2}\\ L_{w}^{\left(2\right)}\left(x,y\right)={\left(x-x_{1}\right)}^{2}{\left(x-x_{2}\right)}^{2}\;\\ L_{w}^{\left(3\right)}\left(x,y\right)={\left(x-x_{1}\right)}^{2}{\left(x-x_{2}\right)}^{2}\; \end{array}\right\}$$

## 4. Free Vibration

The plate strain energy in the kth sub-region is

(24) $$\begin{array}{c} U^{\left(k\right)}=\frac{1}{2}\int_{A_{k}}\left\{N_{x}^{\left(k\right)}\frac{\partial u_{0}^{\left(k\right)}}{\partial x}\right.+N_{y}^{\left(k\right)}\frac{\partial v_{0}^{\left(k\right)}}{\partial y}\;+N_{xy}^{\left(k\right)}\frac{\partial u_{0}^{\left(k\right)}}{\partial y}\;\\ \;\\ \;-M_{x}^{\left(k\right)}\left(\frac{\partial^{2}w_{0}^{\left(k\right)}}{\partial x^{2}}\right)-M_{y}^{\left(k\right)}\left(\frac{\partial^{2}w_{0}^{\left(k\right)}}{\partial y^{2}}\right)\left.-M_{xy}^{\left(k\right)}\left(2\frac{\partial^{2}w_{0}^{\left(k\right)}}{\partial x\partial y}\right)\right\}dA_{k}\; \end{array}$$

where the internal forces and moments in the kth sub-region (*k* = 1, 2, 3, 4) of the functional gradient material plate can be expressed as

(25) $$\left\{\begin{array}{l} N_{x}^{\left(k\right)}\\ N_{y}^{\left(k\right)}\\ N_{xy}^{\left(k\right)} \end{array}\right\}=\left[\begin{array}{ccc} A_{11}^{\left(k\right)} & A_{12}^{\left(k\right)} & 0\\ A_{21}^{\left(k\right)} & A_{22}^{\left(k\right)} & 0\\ 0 & 0 & A_{66}^{\left(k\right)} \end{array}\right]\left\{\begin{array}{c} \frac{\partial u_{0}^{\left(k\right)}}{\partial x}+\frac{1}{2}{\left(\frac{\partial w_{0}^{\left(k\right)}}{\partial x}\right)}^{2}\\ \frac{\partial v_{0}^{\left(k\right)}}{\partial y}+\frac{1}{2}{\left(\frac{\partial w_{0}^{\left(k\right)}}{\partial y}\right)}^{2}\\ \frac{\partial u_{0}^{\left(k\right)}}{\partial y}+\frac{\partial v_{0}^{\left(k\right)}}{\partial x}+\left(\frac{\partial w_{0}^{\left(k\right)}}{\partial x}\right)\left(\frac{\partial w_{0}^{\left(k\right)}}{\partial y}\right) \end{array}\right\}+\left[\begin{array}{ccc} B_{11}^{\left(k\right)} & B_{12}^{\left(k\right)} & 0\\ B_{21}^{\left(k\right)} & B_{22}^{\left(k\right)} & 0\\ 0 & 0 & B_{66}^{\left(k\right)} \end{array}\right]\left\{\begin{array}{c} -\frac{\partial^{2}w_{0}^{\left(k\right)}}{\partial x^{2}}\\ -\frac{\partial^{2}w_{0}^{\left(k\right)}}{\partial y^{2}}\\ -2\frac{\partial^{2}w_{0}^{\left(k\right)}}{\partial x\partial y} \end{array}\right\}$$

(26) $$\left\{\begin{array}{l} M_{x}^{\left(k\right)}\\ M_{y}^{\left(k\right)}\\ M_{xy}^{\left(k\right)} \end{array}\right\}=\left[\begin{array}{ccc} B_{11}^{\left(k\right)} & B_{12}^{\left(k\right)} & 0\\ B_{21}^{\left(k\right)} & B_{22}^{\left(k\right)} & 0\\ 0 & 0 & B_{66}^{\left(k\right)} \end{array}\right]\left\{\begin{array}{c} \frac{\partial u_{0}^{\left(k\right)}}{\partial x}+\frac{1}{2}{\left(\frac{\partial w_{0}^{\left(k\right)}}{\partial x}\right)}^{2}\\ \frac{\partial v_{0}^{\left(k\right)}}{\partial y}+\frac{1}{2}{\left(\frac{\partial w_{0}^{\left(k\right)}}{\partial y}\right)}^{2}\\ \frac{\partial u_{0}^{\left(k\right)}}{\partial y}+\frac{\partial v_{0}^{\left(k\right)}}{\partial x}+\left(\frac{\partial w_{0}^{\left(k\right)}}{\partial x}\right)\left(\frac{\partial w_{0}^{\left(k\right)}}{\partial y}\right) \end{array}\right\}+\left[\begin{array}{ccc} D_{11}^{\left(k\right)} & D_{12}^{\left(k\right)} & 0\\ D_{21}^{\left(k\right)} & D_{22}^{\left(k\right)} & 0\\ 0 & 0 & D_{66}^{\left(k\right)} \end{array}\right]\left\{\begin{array}{c} -\frac{\partial^{2}w_{0}^{\left(k\right)}}{\partial x^{2}}\\ -\frac{\partial^{2}w_{0}^{\left(k\right)}}{\partial y^{2}}\\ -2\frac{\partial^{2}w_{0}^{\left(k\right)}}{\partial x\partial y} \end{array}\right\}$$

where

(27) $$\left(A_{ij}^{\left(k\right)},B_{ij}^{\left(k\right)},D_{ij}^{\left(k\right)})=\int_{-h_{k}/2}^{h_{k}/2}Q_{ij}^{\left(k\right)}(1,z_{k},z_{k}^{2})dz_{k}(i,\;j=1,2,6\right)$$

(28) $$Q_{11}^{\left(k\right)}=Q_{22}^{\left(k\right)}=\frac{E^{\left(k\right)}}{1-\upsilon^{2}},\;\;Q_{12}^{\left(k\right)}=Q_{21}^{\left(k\right)}=\frac{\upsilon E^{\left(k\right)}}{1-\upsilon^{2}},Q_{66}^{\left(k\right)}=\frac{E^{\left(k\right)}}{2(1+\upsilon )}$$

The material properties of the exponential functional gradient material plate vary continuously along the thickness direction. The material parameters of the thickness direction in the above energy equations vary across sub-regions, and the elastic modulus in different sub-regions is expressed as

(29) $$E^{\left(1\right)}=E^{\left(4\right)}=E_{0}e^{\beta z_{1}},\;E^{\left(2\right)}=E_{0}e^{\beta {(z}_{2}+H_{2})},\;E^{\left(3\right)}=E_{0}e^{\beta {(z}_{3}-H_{3})}$$

The total strain energy of the plate is

(30) $$U=U^{\left(1\right)}+U^{\left(2\right)}+U^{\left(3\right)}+U^{\left(4\right)}$$

The kinetic energy of the plate is

(31) $$\left.\begin{array}{c} T=T^{\left(1\right)}+T^{\left(2\right)}+T^{\left(3\right)}+T^{\left(4\right)}\;\\ \begin{array}{c} T^{\left(k\right)}=\frac{1}{2}\int_{-h_{k}/2}^{h_{k}/2}\iint_{A_{k}}\rho^{\left(k\right)}\left[{\left(\frac{\partial u^{\left(k\right)}}{\partial t}\right)}^{2}+{\left(\frac{\partial v^{\left(k\right)}}{\partial t}\right)}^{2}+{\left(\frac{\partial w^{\left(k\right)}}{\partial t}\right)}^{2}\right]dA_{k}dz_{k}\\ \left(k=1,2,3,4\right) \end{array} \end{array}\right\}$$

Here, $\rho^{\left(k\right)}$ is the material density of each sub-zone. Similar to the modulus of elasticity, $\rho^{\left(k\right)}$ can be expressed as

(32) $$\rho^{\left(1\right)}=\rho^{\left(4\right)}=\rho_{0}e^{\beta z_{1}},\;\rho^{\left(2\right)}=\rho_{0}e^{\beta {(z}_{2}+H_{2})},\;\rho^{\left(3\right)}=\rho_{0}e^{\beta {(z}_{3}-H_{3})}$$

Substitute each of the above modal functions $u_{0}^{\left(k\right)}\left(x,y\right),v_{0}^{\left(k\right)}\left(x,y\right),w_{0}^{\left(k\right)}$ into Equations (24)–(26), and (31) to obtain the maximum potential and maximum kinetic energies of each sub-region of the functionally graded material plate during free vibration:

(33) $$\begin{array}{c} U_{max}^{\left(k\right)}=\frac{1}{2}\int_{A}\left\{\left(A_{11}^{\left(k\right)}\frac{\partial u_{0}^{\left(k\right)}}{\partial x}+A_{12}^{\left(k\right)}\;\frac{\partial v_{0}^{\left(k\right)}}{\partial y}-B_{11}^{\left(k\right)}\frac{\partial^{2}w_{0}^{\left(k\right)}}{\partial x^{2}}-B_{12}^{\left(k\right)}\frac{\partial^{2}w_{0}^{\left(k\right)}}{\partial y^{2}}\right)\frac{\partial u_{0}^{\left(k\right)}}{\partial x}\right.\\ +\left(A_{12}^{\left(k\right)}\frac{\partial u_{0}^{\left(k\right)}}{\partial x}+A_{22}^{\left(k\right)}\;\frac{\partial v_{0}^{\left(k\right)}}{\partial y}-B_{12}^{\left(k\right)}\frac{\partial^{2}w_{0}^{\left(k\right)}}{\partial x^{2}}-B_{22}^{\left(k\right)}\frac{\partial^{2}w_{0}^{\left(k\right)}}{\partial y^{2}}\right)\frac{\partial v_{0}^{\left(k\right)}}{\partial y}\;\\ \begin{array}{c} +\left[A_{66}^{\left(k\right)}\left(\frac{\partial u_{0}^{\left(k\right)}}{\partial y}+\frac{\partial v_{0}^{\left(k\right)}}{\partial x}\right)-2B_{66}^{\left(k\right)}\frac{\partial^{2}w^{\left(k\right)}}{\partial x\partial y}\right]\left(\frac{\partial u_{0}^{\left(k\right)}}{\partial y}+\frac{\partial v_{0}^{\left(k\right)}}{\partial x}\right)\;\\ -\frac{\partial^{2}w^{\left(k\right)}}{\partial x^{2}}\left(B_{11}^{\left(k\right)}\frac{\partial u_{0}^{\left(k\right)}}{\partial x}+B_{12}^{\left(k\right)}\;\frac{\partial v_{0}^{\left(k\right)}}{\partial y}-D_{11}^{\left(k\right)}\frac{\partial^{2}w_{0}^{\left(k\right)}}{\partial x^{2}}-D_{12}^{\left(k\right)}\frac{\partial^{2}w_{0}^{\left(k\right)}}{\partial y^{2}}\right)\\ \begin{array}{c} -\frac{\partial^{2}w^{\left(k\right)}}{\partial y^{2}}\left(B_{12}^{\left(k\right)}\frac{\partial u_{0}^{\left(k\right)}}{\partial x}+B_{12}^{\left(k\right)}\;\frac{\partial v_{0}^{\left(k\right)}}{\partial y}-D_{12}^{\left(k\right)}\frac{\partial^{2}w^{\left(k\right)}}{\partial x^{2}}-D_{22}^{\left(k\right)}\frac{\partial^{2}w_{0}^{\left(k\right)}}{\partial y^{2}}\right)\\ \left.-\frac{\partial^{2}w_{0}^{\left(k\right)}}{\partial x\partial y}\left[B_{66}^{\left(k\right)}\left(\frac{\partial u_{0}^{\left(k\right)}}{\partial y}+\frac{\partial v_{0}^{\left(k\right)}}{\partial x}\right)-2D_{66}^{\left(k\right)}\frac{\partial^{2}w_{0}^{\left(k\right)}}{\partial x\partial y}\right]\right\}dA_{k}\; \end{array} \end{array} \end{array}$$

(34) $$T_{max}^{\left(k\right)}=-\frac{1}{2}\omega^{2}\int_{-h_{k}/2}^{h_{k}/2}\iint_{A}\rho^{\left(k\right)}\left[{u_{0}^{\left(k\right)}}^{2}+{v_{0}^{\left(k\right)}}^{2}+{w_{0}^{\left(k\right)}}^{2}\right]dAdz_{k}\;$$

The total energy of the plate containing the crack function gradient material is

(35) $$\Pi^{\left(max\right)}=\sum_{k=1}^{4}\left[U_{max}^{\left(k\right)}+T_{max}^{\left(k\right)}\right]\;$$

The specific expressions (12), (13), and (16) for the displacement function are substituted into the maximum potential energy (32) and the maximum kinetic energy (33) before substituting them into the total energy of the plate (34), and then the corresponding system of equations is obtained by taking the partial derivatives of each of the coefficients to be determined separately:

(36) $$\frac{\partial \Pi^{\left(max\right)}}{A_{ij}^{\left(k\right)}}=0,\;\frac{\partial \Pi^{\left(max\right)}}{B_{ij}^{\left(k\right)}}=0,\;\frac{\partial \Pi^{\left(max\right)}}{C_{ij}^{\left(k\right)}}=0,\;(k=3,\;4,\;5)$$

Here, all the coefficients to be determined in the displacement function are formed into a vector, with the dimension of the vector equal to the total number of all the coefficients to be determined:$\bar{N}$ = 3×3×IJ and $q={\left\{A_{ij}^{\left(1\right)},A_{ij}^{\left(2\right)},A_{ij}^{\left(3\right)},B_{ij}^{\left(1\right)},B_{ij}^{\left(2\right)},B_{ij}^{\left(3\right)},C_{ij}^{\left(1\right)},C_{ij}^{\left(2\right)},C_{ij}^{\left(3\right)}\right\}}^{T}$. The system of Equation (35) can be transformed into

(37) $$\frac{\partial \Pi^{\left(max\right)}}{q_{k}}=0\;(k=l,2,\ldots ,\bar{N})$$

Thus deriving an eigenvalue problem

(38) $$Kq=\omega^{2}Mq$$

The elements in K and M can be represented as

(39) $$K_{ij}=\frac{\partial^{2}U_{max}}{\partial q_{i}\partial q_{j}},\;M_{ij}=\frac{\partial^{2}T_{max}}{\partial q_{i}\partial q_{j}}$$

The eigenvalues and eigenvectors obtained by solving the control Equation (38) correspond to the natural frequencies and modes of the plate, respectively.

## 5. Natural Frequency and Modes

### 5.1. Convergence Analysis and Validation of Results

The geometry of the FGM plate in this section is a/b=2, a/h=100, ν=0.33 and the gradient of the material is $E_{2}/E_{1}=0.2$ and $E_{2}/E_{1}=1$ for both cases. In the Ritz method, as the number of trial function terms increases, the results obtained get closer and closer to the exact solution. First, the convergence and accuracy of the natural frequency results for the intact function gradient material plate are verified. Table 1 examines the first three orders of dimensionless frequencies of the cantilever (CFFF) FGM plate when the number of I and J terms of the trial function in Equations (13), (14), and (17) are taken as 5, 6, 7, and 8. The dimensionless formula is $\omega /\sqrt{D_{0}/\rho_{0}h}$, where $D_{0}=E_{0}h^{3}/12(1-\upsilon^{2})$, and $E_{0}$ and $\rho_{0}$ are the modulus of elasticity and density of the homogeneous material ($E_{2}/E_{1}=$ 1).

It can be seen from Table 1 that the first three orders of frequencies have converged to exact solutions with three significant digits when I×J of the trial function in each displacement field function is taken as 7×7 and 8×8. Therefore, in the next studies in this part, the I×J in the trial functions are taken as 8×8. In order to verify the accuracy of the solutions in this paper, the related literature results are given. It is worth mentioning that there have been no free vibration results for exponential FGM plates in the literature so far. Thus, the comparative results given are from the dimensionless frequencies of crack-containing FGM beams by Yang and Chen in 2008 [42]. For the cantilever boundary conditions, the first- and third-order dimensionless frequencies derived based on the thin plate theory correspond to the first- and second-order dimensionless frequencies of the beam model, respectively, while the second-order mode of the cantilever plate is a torsional vibration, which cannot be considered in the solution of the beam model.

As can be seen from Table 1, the results of this paper are in good agreement with those of the literature, with a relative difference of no more than 3.3%, and these differences occur because the two results are based on plate theory and beam theory, respectively.

In addition to the comparison with the existing literature, ABAQUS 2022 software was applied to analyze the vibration characteristics of plates containing plane cracks. Since the contact effect at the cracked interface needs to be considered, solid units are chosen to model the plate in ABAQUS. Here, the elastic modulus ratio $E_{2}/E_{1}=1$ between the upper and lower surfaces of the FGM plate, and the boundary condition is CCFF. Firstly, the four sub-plates in Figure 1 are modeled separately, and then the sub-plates are linked to form a whole by binding. Hard contacts normal to the contact surfaces of plates ② and ③ in the crack region are set to prevent mutual penetration of the upper and lower sub-plates during vibration. In finite element modeling, the rectangular plate is divided into 0.05 × 0.05 × 0.005 m (length × width × thickness) C3D8R solid cells. From Table 2, it can be seen that the solution of this paper agrees very well with the results of ABAQUS, especially since the error of the first order natural frequency is very small.

The method proposed in this paper is a semi-analytical approach based on the energy method. Compared with the existing finite element method, it avoids the cumbersome meshing process and saves computational costs. As shown in Table 1, an accurate result can be obtained when the basis functions within the plate surface are taken as 8×8. Especially when dealing with functionally graded material plates, where the material parameters vary continuously along the thickness direction, the finite element method requires a sufficient number of meshes to describe such continuous parameter changes to ensure the accuracy of the results.

### 5.2. Effect of Crack Length on Vibration Characteristics of FGM Plates

The size of the crack region is one of the important factors affecting the vibration characteristics of FGM plates. In this paper, it is the crack length that determines the size of the crack region. It is assumed that the crack occurs at the mid-plane of the plate, and the mid-point of the crack region coincides with the mid-point of the plate. The top layer of the FGM plate is made of zirconium oxide ($E_{1}$ = 200 GPa), and the bottom layer is made of aluminum ($E_{2}$ = 70 GPa). Next, the effect of crack length on the natural frequency and modal state of the FGM plate is investigated by considering two boundary conditions, CCFF and CFFF.

**Figure 2:**
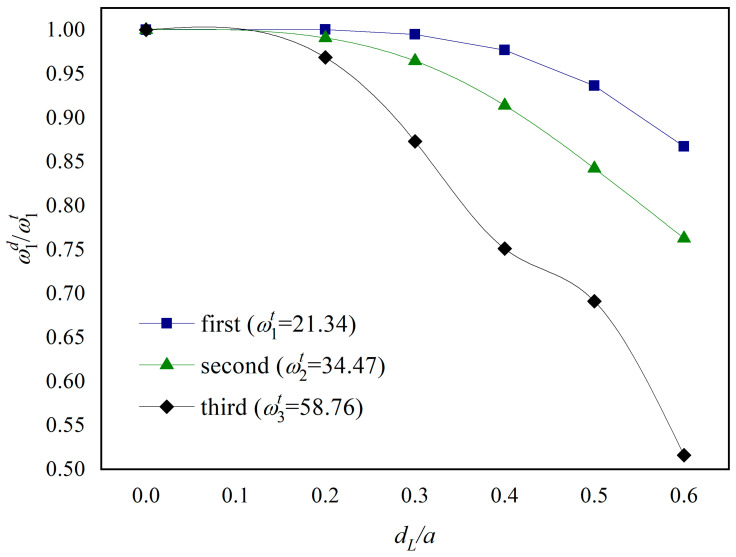
The ratio of natural frequency ($\omega_{1}^{d}/\omega_{1}^{t}$) for an FGM plate with a central mid-plane delamination under CCFF boundary condition.

Figure 2 examines the decrease in the first three orders of the plate’s natural frequencies with increasing crack length for the boundary condition CCFF. The points in the figure are the ratios of the natural frequency of the crack-containing FGM plate to that of the intact FGM plate ($\omega_{i}^{d}/\omega_{i}^{t},\;i=1,2,3$). For comparison, the first-order natural frequency of the intact FGM plate given in the literature [39] and the ratio of the frequencies after the appearance of cracks, $\omega_{i}^{d}/\omega_{i}^{t}$, are given in Figure 2. It can be seen that the natural frequencies of the plate decrease as the length of the cracks increases; the first three orders of the plate’s natural frequencies do not decrease by more than 5% when the length of the cracks is small ($d_{L}/a\leq$ 0.2); the natural frequencies of the plate decrease as the cracks’ length reaches $d_{L}/a=$ 0.6, with the first three orders of natural frequency decreasing by about 15%, 25%, and 50%, respectively, and especially the third-order natural frequency decreasing the fastest.

**Figure 3:**
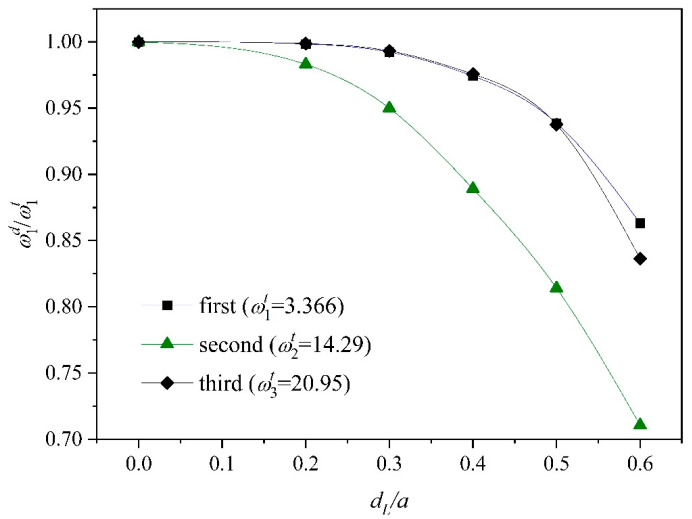
The ratio of natural frequency ($\omega_{1}^{d}/\omega_{1}^{t}$) for a cantilever FGM plate with a central mid-plane delamination.

Figure 3 examines the decrease in the frequency ratio ($\omega_{i}^{d}/\omega_{i}^{t},\;i=1,2,3$) of the FGM plate containing plane cracks to the intact plate under the cantilever (CFFF) boundary condition. It is seen that the natural frequency of each order decreases as the crack length increases. Among them, both the first and third orders decrease by about 85% at a crack length of $d_{L}/a=$ 0.6. The second-order frequency decreases faster and has the largest magnitude. The second-order frequency decreases by nearly 30% at a crack length of $d_{L}/a=$ 0.6.

**Figure 4:**
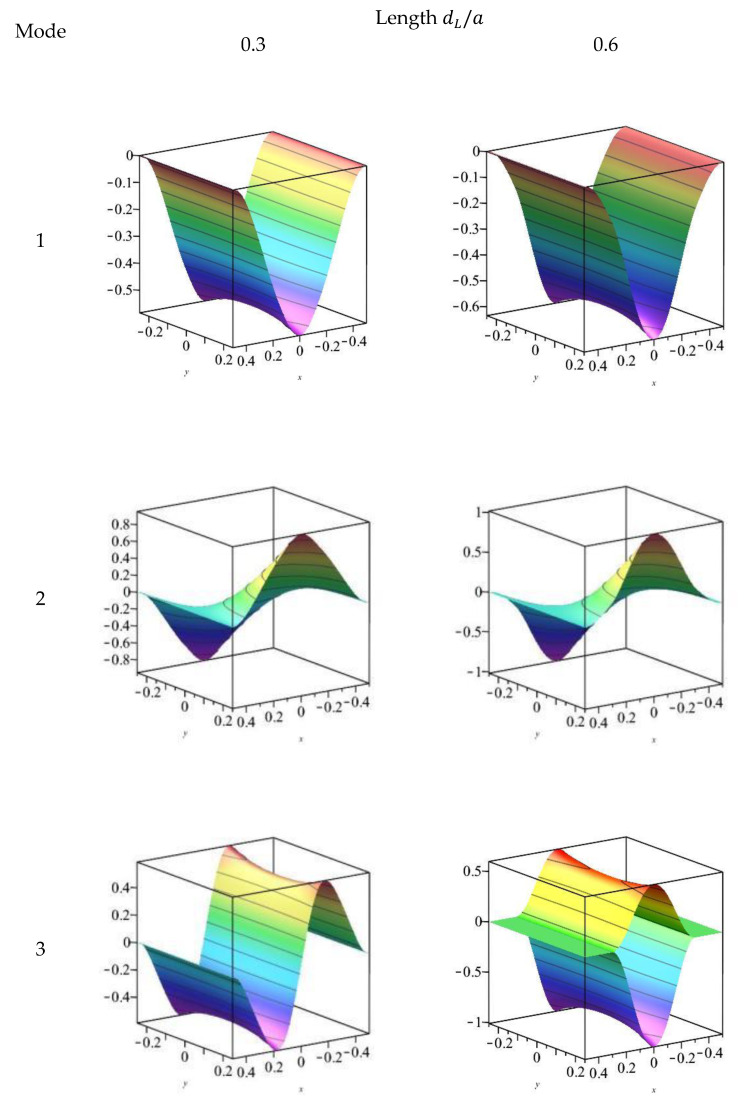
The first three modes of *w* for an FGM plate with different delamination lengths under CCFF boundary condition.

**Figure 5:**
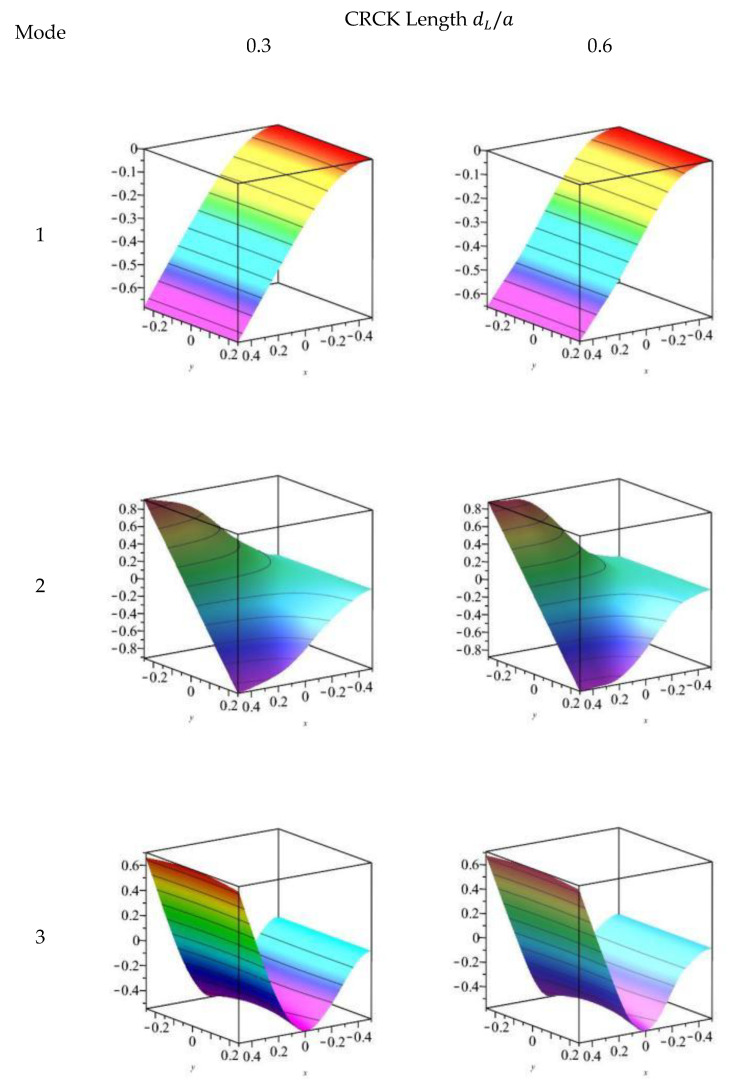
The first three modes of *w* for an FGM plate with different delamination lengths under CFFF boundary condition.

The first three orders of modes for the transverse displacement w of the FGM plate containing a plane crack are given in Figure 4 and Figure 5 for the CCFF and CFFF boundary conditions, respectively. At crack length $d_{L}/a=$ 0.3, the first three order modes of the cracked plate are close to the intact plate for both boundary conditions. At $d_{L}/a=$ 0.6, the third-order modes of the transverse displacement ω for the CCFF boundary condition changed significantly: the displacements at the top and bottom of the cracked region were in phase with each other, forming an opening; since the stiffness of the lower surface of the FGM plate was less than that of the upper surface, the displacement of the lower plate was larger than that of the top. No significant change in the modal state was observed in the other cases.

### 5.3. Effect of Crack Location on Vibration Characteristics of FGM Plates

In actual structures, the location of the crack damage appearance is often uncertain. The randomness of external loads and the complex stress distribution of the structure may lead to cracks appearing at arbitrary locations, so it is necessary to study the effect of crack location on the vibration characteristics of the structure. In this section, it is assumed that the in-plane crack length of the FGM plate is kept constant at $d_{L}/a=$ 0.4, and the natural frequencies and modes are solved by changing the crack location $d_{c}$.

Table 3 examines the effect of crack location on the natural frequencies of the FGM plate with both the CCFF and CFFF boundary conditions considered. For the CCFF plate, the closer the in-plane location of the crack is to the center line of the plate (x=0), the larger the first two orders of the natural frequencies are and the smaller the third-order frequency is. Conversely, the closer the location of the crack is to the ends of the plate, the smaller the first two orders of the natural frequencies are and the larger the third-order frequency is. Since the structure and boundary conditions of the plate are symmetrical in the plane, the variation of the natural frequency also has some symmetry characteristics. Combined with the modes of the intact CCFF plate in the previous section, the nodal lines of the first two modes are at the solidly supported edges of the plate, while the third-order mode has an additional nodal line falling at the center line of the plate. It can be concluded that the closer the crack region is to the nodal line of the mode, the greater the corresponding decrease in the natural frequency. For the CFFF plate, the closer the in-plane location of the crack is to the fixed end of the plate (x=−0.5a), the smaller the first two orders of natural frequency are. Conversely, the closer the location of the crack is to the free end of the plate, the larger the first two orders of natural frequency are. The variation of the third-order frequency is more complicated and is determined by the boundary conditions of the plate and the nodal lines of the modes.

**Figure 6:**
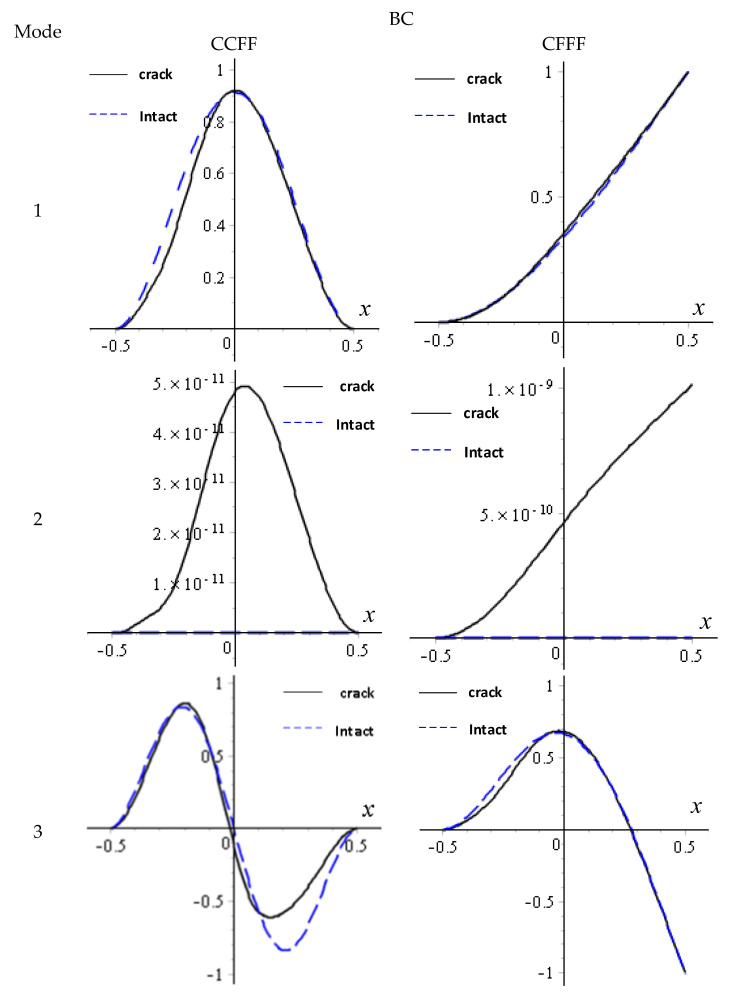
The first three modes of *w* for a delaminated FGM plate with delamination parameters $d_{c}/a=$ −0.1 and $d_{L}/a=$ 0.4.

Figure 6 gives the first three orders of modal curves of w under the two boundary conditions when the position of the crack surface is ${\;d}_{c}/a=-0.1$ (${\;d}_{L}/a=-0.4$). Firstly, the obtained modes are normalized, which means the modal displacement w is divided by its maximum value $w_{max}$, and taking one of the curves with y=0 in the modes, the modal curves are obtained. For comparison, the corresponding modes of the intact plate are given by blue dashed lines in the figure. It can be seen that a change in position within the cracked surface destroys the position of the point of maximum amplitude in the modes and the symmetry of the modes; for the intact CCFF and CFFF boards, the nodal line of their second-order modes just falls on y=0, where the amplitude is almost equal to 0. However, after the appearance of the crack in the plate, the amplitude at the y=0 position of the mode experienced a slight change. From the magnitude of the change, it can be determined that the nodal line is still near y=0.

### 5.4. Natural Frequency Study of Cracked FGM Plates with Different Material Gradients

**Figure 7:**
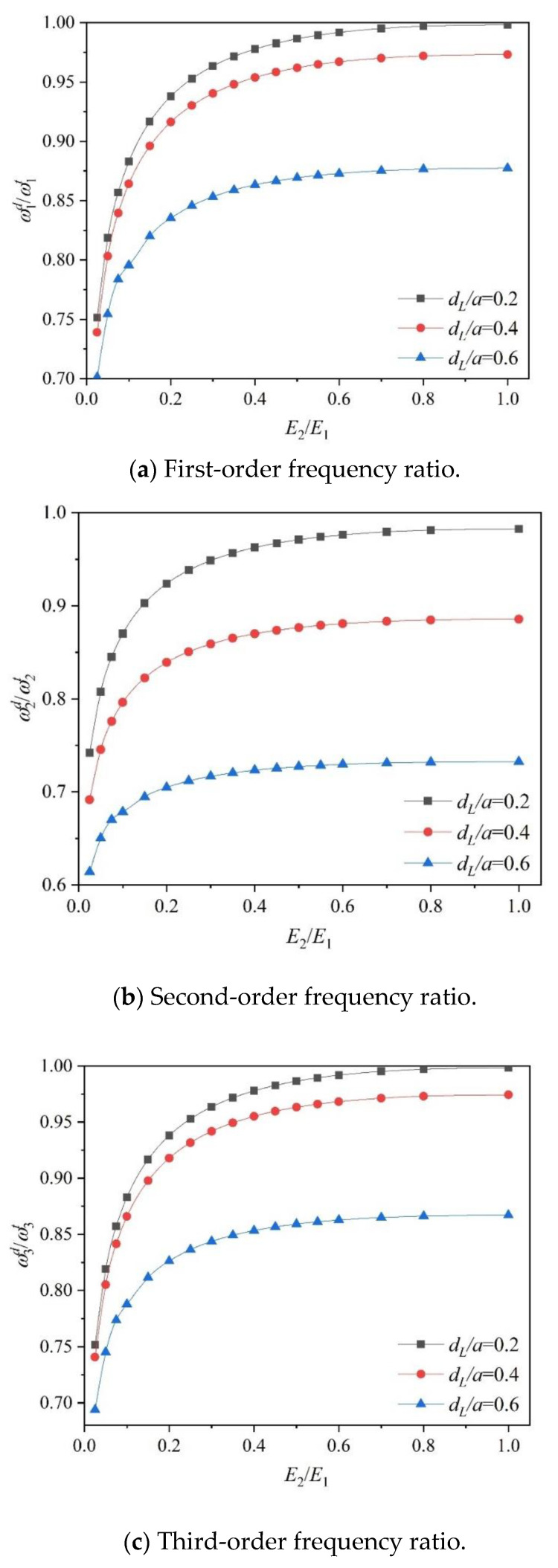
The frequency ratios versus Young’s modulus ratio with various delamination lengths under CFFF boundary conditions ($d_{c}/a=0$).

Figure 7 examines the effect of material gradient change on the first three orders of natural frequency of an FGM plate with plane cracks under CFFF boundary conditions. Here, the crack location of the plate is ${\;d}_{c}=0$, and the lengths are $d_{L}/a=0.2$, 0.4, and 0.6, respectively, and $E_{2}/{\;E}_{1}$ on the upper and lower surfaces of the FGM plate increases from 0.05 to 1. The vertical coordinate of the figure is the ratio of the natural frequencies of the delaminated FGM plate and the intact plate, with $E_{2}/{\;E}_{1}=1$. It can be seen that with an increase in $E_{2}/E_{1}$, the natural frequency of the FGM plate increases, and the gap of natural frequency under different crack lengths increases; with an increase in crack length, the effect of material gradient property on natural frequency is weakened, especially for the second-order natural frequency.

## 6. Conclusions

In this study, a plane-crack-containing functionally graded material (FGM) plate is divided into four sub-zones using the zone method. The kinetic and potential energies of each sub-zone are derived by applying the thin plate theory in conjunction with the von Kármán type geometric nonlinear strains. Subsequently, the trial functions of the displacement field are constructed based on the boundary conditions and deformation coordination conditions of each sub-zone. The free vibration characteristics of the FGM plate are then obtained using the Ritz method under the assumption of small deformation. By analyzing the natural frequencies and mode shapes of the FGM plate under various crack and gradient parameters, the following conclusions are drawn:

(1) As the length of the plane crack increases, the influence of the gradient parameter on the natural frequency diminishes. Specifically, a longer crack reduces the sensitivity of the natural frequency to variations in the gradient parameter.
(2) The effect of crack location on the natural frequency is governed by both the boundary conditions of the FGM plate and the nodal lines of the mode shapes for each order. Moreover, the presence of a plane crack alters the nodal lines and symmetry of the mode shapes. This indicates that the crack location interacts with the boundary conditions and mode characteristics to influence the vibration behavior of the plate.
(3) During free vibration, the upper and lower parts of the FGM plate in the plane crack region exhibit a deviation from each other, resulting in an opening behavior. This deviation is further influenced by the material gradient, causing the upper and lower parts of the opening region to have different displacements and exhibit asymmetry in the thickness direction. This phenomenon highlights the significant impact of material gradient on the vibration response of a cracked FGM plate.

## Figures and Tables

**Table 1:** Convergence of non-dimensional frequency $\omega /\sqrt{D_{0}/\rho_{0}h}\;$ of an intact FGM plate under cantilever boundary condition.

$E_{2}/E_{1}$	Mode	*I × J*				Yang [40]	Relative Error
		5 × 5	6 × 6	7 × 7	8 × 8		
0.2	1	3.224	3.220	3.217	3.216	3.30	2.5%
	3	20.04	20.03	20.02	20.02	20.70	3.3%
1	1	3.431	3.427	3.424	3.423	3.52	2.8%
	3	21.33	21.32	21.31	21.31	22.03	3.3%

**Table 2:** The first three-order natural frequency (Hz) for a CCFF FGM plate ($E_{2}/E_{1}=$1) with a central mid-plane delamination ($d_{L}$/a = 0.5).

Method	Frequency (Hz)		
	1	2	3
This paper	84.45	122.40	170.39
ABAQUS	84.20	123.46	168.65
relative error	0.30%	0.87%	1.0%

**Table 3:** The non-dimensional frequency $\omega /\sqrt{D_{0}/\rho_{0}h}\;$ of an FGM plate with a mid-plane delamination having different locations, $d_{c}$.

BC	Mode	$\mathrm{Position}\;d_{c}/a$				
		−0.1	−0.05	0	0.05	0.1
CCFF	3	48.79	46.22	44.60	46.22	48.79
	2	31.21	31.69	31.84	31.69	31.21
	1	20.04	20.79	21.07	20.79	20.04
CFFF	3	19.37	20.00	20.45	20.60	20.47
	2	12.49	12.62	12.70	12.93	13.12
	1	3.274	3.278	3.279	3.294	3.305

## Data Availability

The original contributions presented in the study are included in the article, further inquiries can be directed to the corresponding authors.

## Abbreviations

symbol	description
a	the length of the plate
b	the width of the plate
$d_{L}$	the length of the crack
$d_{c}$	the distance between the mid-point of the planar crack and the mid-point of the plate
E	modulus of elasticity of the FGM plate
ρ	density of the FGM plate
β	the gradient parameter of the material
$E_{1}$ $/\upsilon_{1}$ $/\rho_{1}$	modulus of elasticity, Poisson’s ratio, and density at the upper surfaces of the plate
$E_{2}$ $/\upsilon_{2}$ $/\rho_{2}$	modulus of elasticity, Poisson’s ratio, and density at the lower surfaces of the plate
FGM	functionally graded material
CFFF	For a plate solidly supported at $x\;=\;-\frac{a}{2}$ with other sides free
CCFF	x=−a/2 and x=a/2 are fixed and the other two sides are free
